# Effects of Electrode Materials on Electron Transport for Single-Molecule Junctions

**DOI:** 10.3390/ijms24087277

**Published:** 2023-04-14

**Authors:** Mong-Wen Gu, Chun-hsien Chen

**Affiliations:** Department of Chemistry and Center for Emerging Materials and Advanced Devices, National Taiwan University, Taipei 10617, Taiwan

**Keywords:** electrode materials, molecular electronics, scanning probe microscopy, single-molecule junction conductance

## Abstract

The contact at the molecule–electrode interface is a key component for a range of molecule-based devices involving electron transport. An electrode–molecule–electrode configuration is a prototypical testbed for quantitatively studying the underlying physical chemistry. Rather than the molecular side of the interface, this review focuses on examples of electrode materials in the literature. The basic concepts and relevant experimental techniques are introduced.

## 1. Introduction

Electron transfer is an elementary process associated with many physical or chemical phenomena, such as synthesis reactions of molecules, the operation of electronic devices, conversion of solar energy, electrocatalysis, and corrosion. In recent decades, it has become possible to carry out measurements of single-molecule junction conductance. The understanding of electron transport across electrode–molecule–electrode (EME) junctions as attracted enormous research interest due to the following reasons. Firstly, the typical size of single molecules is at the level of nanometers, making them ideal building blocks for nanoelectronic devices. Secondly, the physical and chemical properties of molecules are associated with their chemical structures. Hence, the functions of molecule-based devices can be tailored through the rational design of molecular structures [1]. Thirdly, as an electronic device shrinks to the single-molecule level, quantum effects become significant, and many interesting phenomena will be encountered, including quantum interference [2,3], inelastic tunneling [4], and spin dynamics [5]. Additionally, some research groups have shown that single-molecule devices exhibit promising applications, such as those in topological insulators [6] and reaction sensors [7].

**Figure 1:**
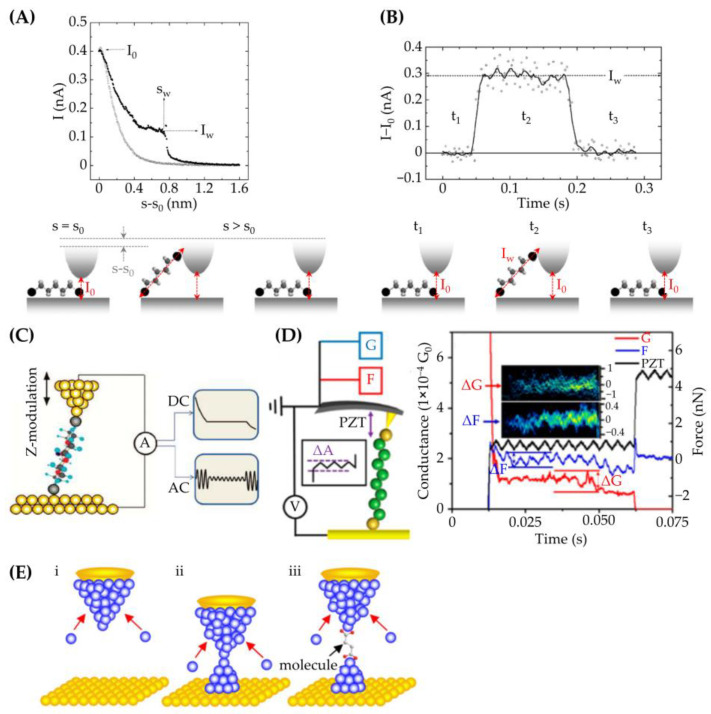
Schematic illustrations of experimental approaches developed with a scanning probe microscope (SPM) for the characterization of single-molecule junctions. (**A**) The SPM i(s) mode is employed to monitor the dependence of the tunneling current on the displacement of the z-axis piezo. The tip approaches the substrate until a pre-set current value (I_0_) is reached. The tip is then retracted. If a molecule is in contact with both the tip and the substrate, as the distance of tip–substrate separation (s − s_0_) increases, a current plateau (I_w_) will develop due to the electron tunneling mediated by the molecule. Otherwise, there will only be the background current of the through-space tunneling (e.g., the gray trace). (**B**) The SPM i(t) mode, in which the tip is fixed at a certain tip–substrate separation, is used to detect the stochastic events of the formation of molecular junctions. A current jump from I_0_ to I_0_ + I_w_ indicates that a molecule is bridged between the tip and the substrate, where I_0_ and I_w_ denote the tunneling currents through the tip–substrate gap and the molecule, respectively. The event of a current drop from I_0_ + I_w_ to I_0_ indicates the breakdown of a molecular junction. (**C**) STM-BJ experiments were integrated with an AC voltage source that modulated the movement of the z-axis piezo. The detected current was deconvoluted into the DC and AC parts, respectively, corresponding to the current through the molecule and current fluctuation due to the modulation. The amplitude of the AC part indicates whether a molecule is bridged between the electrodes. (**D**) AFM-BJ experiments were integrated with an AC modulation. This approach was employed to analyze the cross-correlation between the force and conductance. (**E**) EC-STM is used to prepare electrodes for EME junctions. i: Atoms are electrodeposited on the tip from metal ions in the solution. ii: The tip is moved toward the substrate until the monitored current reaches a pre-set value. Subsequently, a pulsed voltage is applied to the piezoelectrics, leading to a further movement of the tip toward the substrate. Then, the atoms deposited on the tip are transferred to the substrate. iii: The tip is retracted from the substrate, during which the metallic contact is broken and a molecular junction is formed. The figures are adopted from [12,13,14,27].

Well-tested platforms for conductance measurements of molecular junctions are necessary to increase our understanding of transport behaviors in single-molecule devices. Presently, the most popular approach is the break-junction (BJ) technique, in which a nanoscale metallic contact is mechanically separated [8,9] and developed into a nano-gap to host molecules. Upon further opening the gap, the number of molecules bridging the electrodes is reduced, and a single-molecule EME junction may eventually be formed. The break-junction technique has gained a great success in characterizing the transport behavior of EME junctions. The first platform, which was reported by Reed and co-workers [8], had a gold wire fixed on a flexible substrate and immersed in tetrahydrofuran solution containing 1 mM benzenedithiol. After bending the substrate, the gold wire was stretched until it broke into two parts. A single molecule hence made contact between the electrodes and formed an EME junction. Alternatively, Tao and co-workers employed a scanning tunneling microscope (STM) to carry out BJ experiments [9]. An STM contains a key component called a piezoelectric actuator that controls the movement of a tip to scan over a substrate. The deformation of the piezoelectric actuator depends on the electronics of a feedback loop in response to the monitored tunneling current between the tip and the substrate. This enables the tip to repeatedly move forward and backward from the substrate. Conductance plateaus with multiple integers of G_0_ (conductance quantum, 1 G_0_ ≈ 77.6 μS) were observed when the tip was retracted away, exhibiting the features of a quantum-point contact of Au atoms. Subsequently, the Au contact was broken and a nano-gapped junction was formed and made available to host molecules. The workflow of the STM-BJ technique provides an efficient way to obtain reliable experimental results. Furthermore, the junction spacing can be facilely changed through the deformation of the piezoelectric component driven by an external bias. Hence, via a pre-programmed voltage waveform, a desirable routine of spacing changes can be delivered to meet an experimental design. For example, Haiss and co-workers developed approaches called STM i(s) and i(t) modes (Figure 1A,B), where s and t, respectively, denote the displacement of the z-axis piezo and time [10,11,12]. The STM i(s) mode allows the operator to monitor the dependence of the tunneling current on the separating spacing of electrodes and tilt angles of the molecular backbone. On the other hand, the STM i(t) mode allows the operator to observe the time evolution of the tunneling current due to stochastic events in the electrode gap.

Tao and co-workers employed a modulation technique to subtract the background of vacuum tunneling from the measured conductance [13] (Figure 1C). Similarly, Xu and co-workers integrated an atomic force microscope (AFM) with the modulation technique to investigate the cross-correlation between the force and conductance [14,15] (Figure 1D). Chen and co-workers incorporated a conductive AFM with a tactile feedback system to configure and characterize single-molecule junctions in a way in which molecular junctions could be manually maintained for an extended period of several minutes [16]. This experimental approach enabled experiments demanding long acquisition times (e.g., measurements of i-V characteristics). Chang’s group integrated both the i(s) and i(t) modes of STM to monitor the evolution of a BJ process [17]. This hybrid approach reduced the stochastic effects of the formation of molecule–electrode contacts on the molecular conductance and resulted in conductance histograms with peaks narrower than those of typical BJ techniques. Tao and co-workers demonstrated BJ measurements of oligo(phenylene-ethynylene)s with an electrochemical STM (EC-STM) [18]. This approach enabled the control of the electrode Fermi level (E_Fermi_) relative to the energy level of the frontier molecular orbital (E_FMO_). In electrochemical environments, the transport behavior of a single molecule can be tuned with an external gate electrode, resembling a conventional transistor [19,20,21,22,23,24,25]. Mao and co-workers applied an EC-STM to in situ deposit foreign metals (e.g., Ag or Cu clusters) on Au(111) substrates [26,27] (Figure 1E). This technique provided an opportunity to systemically investigate the molecular conductance of electrodes of various materials.

This review article aims at the role played by electrodes in electron transport across molecular junctions. Experiments were carried out in solutions with molecules of interest and under ambient conditions unless otherwise noted. The basic principles of electron transport are discussed, followed by an introduction to an experimental technique called transition voltage spectroscopy (TVS), which was employed to investigate electrodes’ effects on molecular conductance. The studies of junction conductance on three types of metallic materials are reported, including noble (e.g., Pt, Pd, Ag, and Cu), magnetic (e.g., Fe, Co, and Ni), and bimetallic ones. Finally, research efforts with carbon-based materials, including carbon nanotubes and graphene, are reported.

## 2. Effects of Electrodes on Molecular Conductance

### 2.1. Basic Principles

**Figure 2:**
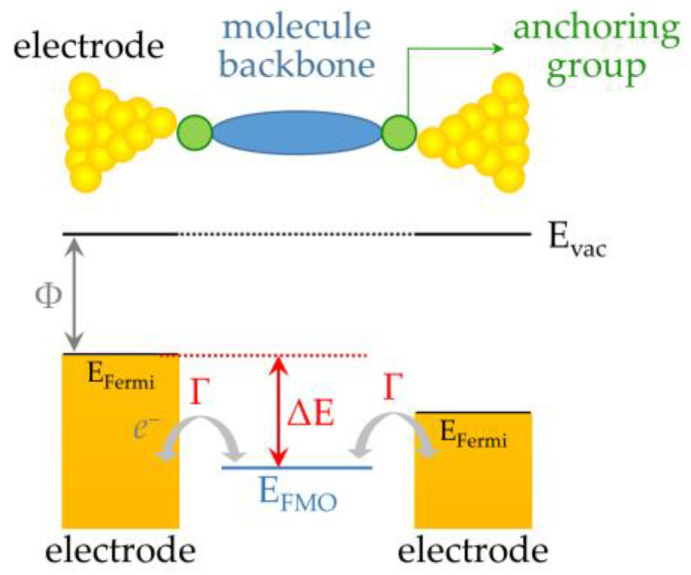
Scheme of a single-molecule junction with a molecule bridged between a pair of electrodes. The lower panel depicts the electrode–molecule coupling, Γ, and the degree of energy-level alignment, ΔE, namely, the difference between E_Fermi_ and E_FMO_, where E_Fermi_ and E_FMO_ denote the energy of the Fermi level and frontier molecular orbital, respectively.

Figure 2 presents a general sketch of a single-molecule junction in which the electrode–molecule contacts are furnished via anchoring groups. Additionally displayed in Figure 2 is a simplified model to illustrate electron transport across a single-molecule junction, which is known as the single-level model [28,29]; here, the behavior of electron transport is mainly dominated by the frontier molecular orbital (FMO) of the molecule, and two parameters are considered, namely, energy-level alignment (ELA ≈ΔE) and the electrode–molecule coupling (Γ). The ELA describes the energy-level difference between the FMO of the molecule and the Fermi energy of the electrodes. Γ evaluates the coupling strength between the electrodes and the transporting channel, e.g., the FMO in the example of Figure 2.

The abovementioned single-level model shows that the ELA and Γ are important parameters for describing the transport properties. Both the ELA and Γ are associated with molecular properties, such as E_FMO_ and the affinity for electrodes. Accordingly, to increase transport efficiency, one can design promising molecular structures to pair with suitable electrode materials. On the other hand, tuning the electrode properties is a possible approach to facilitating transport efficiency. Electrodes play an equally important role to that of the molecules, even though candidates of the electrode materials are rather limited, so relevant research reports are far fewer than those on molecular structures. Electrodes are not only reservoirs of electrons, but also have significant effects on the E_FMO_ value of a molecule. When a molecule adsorbs onto an electrode, the molecular orbitals are perturbed by the electrode [30]. This effect is formulated by the Newns–Anderson model and is termed self-energy, Σ where Σ=Δ+iΓ. The real (Δ) and imaginary (Γ) parts of the self-energy correspond to the shift and the broadening of E_FMO_, respectively [30]. With the incorporation of the d-band theory developed by Nørskov and co-workers, Newns–Anderson model provides a strategy for tuning the self-energy through rational design of the electrode materials [31,32,33,34]. In addition to the energy-level shifting and broadening, electrodes may induce a phenomenon called partial charge transfer across the molecule–electrode interface. The occupation number of the FMO is not necessary an integer, which might result in the formation of interfacial dipoles and the effects of Fermi-level pinning [35]. Due to the above-mentioned effects, understanding the effects of electrodes on single-molecule junctions is essential.

### 2.2. Characterization Methods

Experimentally, the degree of ELA and values of Γ for a single-molecule junction can be quantitatively determined with the semi-logarithm plot of the measured conductance versus the lengths of homologous molecules ($G=G_{n=0}e^{{–\beta }_{n}n}$) [36], where the slope and the intercept of this semi-logarithm plot correspond to the tunneling constant (β*_n_*) and the contact conductance (G*_n_*_=0_) of the molecules. β*_n_* represents the transport efficiency across the molecular backbone, which generally indicates the degree of ELA. G*_n_*_=0_ represents the coupling strength between the molecule and electrodes and is, thus, associated with the degree of Γ.

**Figure 3:**
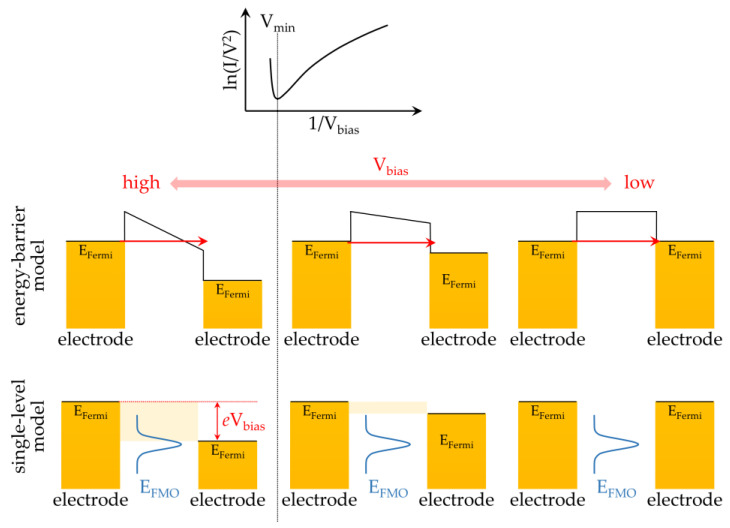
Interpretation of the trace of transition voltage spectroscopy (TVS). An important feature of a TVS spectrum is its minimum point (V_min_), which is described by the energy-barrier model (**middle** panels) and single-level model (**bottom** panels) for electron transport across an ensemble junction or a single-molecule junction, respectively. For the **middle** panels, the molecular ensemble is viewed as an energy barrier whose shape is determined by the applied bias (V_bias_). The shape of the energy barrier changes from a trapezoidal one to a triangular one when V_bias_ > V_min_, suggesting the transition of the transport mechanism from tunneling to field emission (or Fowler–Nordheim tunneling). For the **bottom** panels, the transport is dominated by the frontier molecular orbital and is described by a transmission peak. When V_bias_ > V_min_, the bias window (beige-shaded area) covers a certain fraction of the proximal transmission peak. The value of V_min_ increases as the position of E_FMO_ shifts away from the Fermi level (E_Fermi_), showing that V_min_ is a parameter indicating the degree of ELA.

Another tool for characterizing the ELA is TVS (Figure 3), which was first performed by Frisbie and co-workers [37]. A TVS spectrum is derived by transforming the measured *i*−V characteristics of single-molecule junctions into the plot of ln(*i*/V^2^) versus 1/V. In TVS spectra, there is a minimum point at a specified voltage, which is usually termed V_min_ or V_trans_ (Figure 3). The values of V_min_ typically indicate the transition of transport mechanisms from direct tunneling to field emission. In Frisbie’s work, the model system was a self-assembled monolayer (SAM) of organic molecules sandwiched between electrodes. For electrons, the SAM is a tunneling barrier whose shape is decided by the applied bias. In the small bias regime, the shape of the barrier is trapezoidal and the transport mechanism is tunneling. When the bias exceeds V_min_, the shape of the barrier becomes triangular and the transport mechanism, thus, turns into field emission. Accordingly, the value of V_min_ depends on the height of the barrier.

The concept of TVS is considered applicable to single-molecule junctions, even though Frisbie’s work was demonstrated by monolayers. With a simple resonant tunneling model, Araidai and Tsukada [38] showed that the TVS spectra of a single-molecule junction displayed features similar to those of ensemble junctions. Noteworthily, the physical meaning of V_min_ for single-molecule junctions was interpreted differently from that of ensemble ones. In Araidai and Tsukada’s scenario, the turning point of a TVS trace occurred as the bias window (i.e., V_min_) covered a certain fraction of the proximal transmission peak. A smaller V_min_ typically represents a smaller difference between the FMO level and the Fermi level and, hence, a better degree of ELA [38]. Many reports in the literature have shown that ELA determined through TVS is in good agreement with that determined through ultra-violet photoemission spectroscopy (UPS) [39,40,41], demonstrating that TVS is a practical tool for investigating the degree of ELA.

## 3. Metallic Material Electrodes

Metals are common materials for the study of electron transport across an EME junction. The ductility of metals renders them potential candidates for delivering BJ experiments, and thus, metal-based EME junctions can be relatively facilely obtained. This feature enables the collection of thousands of conductance traces that cover many molecular conformations, molecule–electrode binding configurations, and restructured apical geometries of electrodes in EME junctions. The measured traces are, therefore, subject to statistical analysis. In the routine of BJ measurements, upon the breakage of the quantum-point contact, a phenomenon called “snap-back” occurs. It suddenly opens up a gap between electrodes, and correspondingly, the measured current abruptly drops by orders of magnitude. The snap-back magnitude of Au electrodes is estimated to be 5–7 Å [42,43,44,45,46], which creates a well-defined and sharp drop in the conductance and makes the conductance plateau of the bridged molecule distinct from that of the Au contact. Because of this feature and its resistance to oxide formation, Au is a very popular electrode material for the study of single-molecule conductance.

The distribution of a conductance peak is typically over an order of magnitude due to the abovementioned EME structures. The preparation of two-dimensional conductance histograms by plotting the junction conductance against the electrode displacement confers a better resolution and alleviates the challenges in peak interpretation [47]. The groups of Halbritter and Venkataraman developed an advanced method based on cross-correlation analysis [48]. Through the analysis of features in conductance traces, one can reveal information about the evolution of an EME junction and explore detailed mechanisms of electron transport.

Table 1 presents the results from the literature for single-molecule junction conductance for α,ω-alkanes (X-(CH_2_)*_n_*-X). The HOMO-LUMO gaps of alkanes are large, and their E_FMO_ values are a few eVs away from the electrode Fermi level. Therefore, the difference in the junction conductance is mainly ascribable to the effects of interfacial couplings (i.e., Γ). Thiol-terminated molecules have strong coupling with Au electrodes. The contact conductance (G*_n_*_=0_) is about 0.1–0.5 G_0_ [49,50], and the rupture force required to break the contact is about 1.5 nN [51]. The thiol–gold contact features exceptional transport efficiency and mechanical stability among headgroup–electrode pairs, enabling the study of many interesting phenomena, such as quantum interference [2], current blockades [52], and on-surface reactions [53].

### 3.1. Pt, Pd, Ag, and Cu Electrodes

The group of van Ruiteneek examined the stretched lengths prior to contact breaking for materials of Pt, Pd, Ag, and Cu, along with Ir, Rh, and Au, via mechanically controllable break junctions at 4.2 K [61]. The results showed that Ir, Pt, and Au formed freely suspended chains of atoms, while Rh, Pd, Ag, and Cu did not form a long monoatomic chain. The difference was explained by the relativistic effects for Ir, Pt, and Au, which are 5d elements. To balance the energy of the contracted s shell, the interactions between the d orbitals of under-coordinated atoms are stronger and render shorter bond lengths than those of their bulk forms.

**Figure 4:**
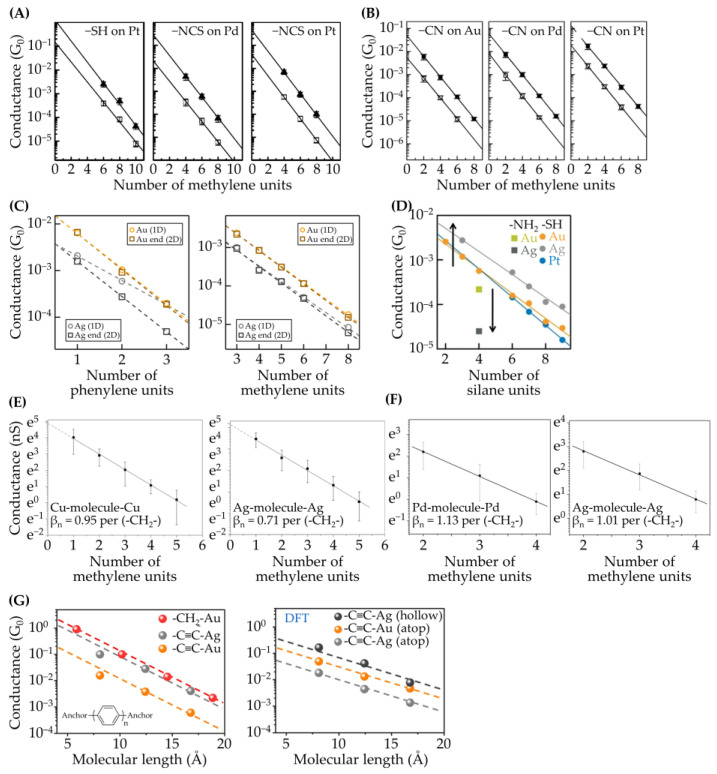
Effects of electrode materials on junction conductance. The transport efficiency across the molecule–electrode contacts is evaluated according to the values of contact conductance (G*_n_*_=0_), which are determined by the intercept in the plot of conductance versus molecular length. Electrodes: (**A**,**B**) Au, Pd, and Pt, (**C**) Au and Ag, (**D**) Au, Ag, and Pt, (**E**) Cu and Ag, (**F**) Pd and Ag, and (**G**) Au and Ag. Model compounds: (**A**) α,ω-alkane-based dithiols and diisothiocyanates (X-(CH_2_)*_n_*-X, where X = −SH, −SCN, and *n* = 4, 6, 8), (**B**) α,ω-alkane-based dinitriles (NC-(CH_2_)*_n_*-CN, *n* = 4, 6, 8) [36], (**C**) amine-terminated oligophenyls (H_2_N-(C_6_H_4_)*_n_*-NH_2_, *n* = 1–3) and α,ω-alkane-based diamines (H_2_N-(CH_2_)*_n_*-NH_2_, *n* = 3–6), (**D**) amino- and thiol-terminated permethyloligosilanes (H_2_N-(Si(CH_3_)_2_)_4_-NH_2_; HS-(Si(CH_3_)_2_)*_n_*-SH, *n* = 2–4, 6–9), (**E**,**F**) α,ω-alkane-based dicarboxylic acids (HO_2_C-(CH_2_)*_n_*-CO_2_H, *n* = 1–5), and (**G**) acetylene-terminated oligophenylenes (HCC-(C_6_H_4_)*_n_*-CCH, *n* = 1–3). The figures are adopted from [36,50,55,65,66,67,71].

Chen and co-workers studied the junction conductance of a series of α,ω-alkane-based dithiols [50], diisothiocyanates [50], and dinitriles [36] (X-(CH_2_)*_n_*-X, where −X = −SH, −SCN, −CN, and *n* = 4, 6, 8) on Au, Pt, and Pd electrodes (Figure 4A,B). The junction conductance of Pt and Pd electrodes was about 1.3–2.6 times that of Au. The fitting of the Simmons formula to the *i*−V_bias_ curves yielded barrier heights (Φ_Β_) of 1.3–1.4 eV for these EME junctions. Their tunneling decay constants (β*_n_*) all fell in the range of 0.96–1.08 (per methyl unit). Their similar Φ_Β_ and β_n_ values caused the disparate junction conductance to be unattributable to their E_FMO_ values. The values of G*_n_*_=0_ of these anchoring groups on Pt and Pd electrodes were 1.4–5.3 times larger than those on Au electrodes. Through further investigation of the headgroup–electrode interactions by using density functional theory (DFT) calculations and molecular orbital analysis (AOMix) [62,63], the superior contact conductance for Pt and Pd was attributed to their d-orbital contributions.

Venkataraman and co-workers studied a single-atom contact under ambient conditions. In addition to the conductance peak of a single Ag atom contact (Ag-SAC) at 1 G_0_, the histogram exhibited oxygen-involved features at about 1.3 G_0_ and 0.4 G_0_ [64] due to the reaction of Ag with ubiquitous oxygen. Ag-O-Ag configurations in which an oxygen atom bridges in parallel with Ag-Ag (AgO-P) or in series to form Ag-O-Ag junctions (AgO-S) were proposed. AFM measurements showed rupture forces of 1 nN, 1.7 nN, and 0.8 nN for the Ag-SAC, AgO-P, and AgO-S junctions, respectively [64]. Regarding the snap-back behavior of Ag electrodes, a gap of ~5 Å was created with the first 10 μs and finally became ~1.3 nm, which was ascribed to the restructured apical geometries due to the large diffusion constant [55].

For EME junctions of amine-terminated oligophenyls (H_2_N-(C_6_H_4_)*_n_*-NH_2_, *n* = 1–3) and α,ω-alkanes (H_2_N-(CH_2_)*_n_*-NH_2_, *n* = 3–6), the conductance on Ag electrodes was about 1/3–1/4 of that on Au electrodes (Figure 4C) [55]. This discrepancy was ascribed to the difference in their work functions. Ag has a smaller work function than Au and, thus, has a larger energy difference of |E_Fermi_ − E_HOMO_|. Accordingly, the smaller work function of Ag leads to degrees of ELA and transport efficiency that are inferior to those of Au electrodes. However, the junction conductance of amino- and thiol-terminated permethyloligosilanes (H_2_N-(Si(CH_3_)_2_)_4_-NH_2_; HS-(Si(CH_3_)_2_)*_n_*-SH, *n* = 2–4, 6–9) is quite intriguing (Figure 4D). For the former, Ag electrodes confer a smaller junction conductance than that of Au, which is consistent with the explanation of ELA. However, the junctions of thiol-terminated molecules on Ag electrodes were found to be more conductive than those on Au electrodes [65]. The authors suggested that the molecular conductance does not depend solely on the degree of ELA, and the headgroup–electrode couplings should be taken into account.

Mao, Zhou, and their colleagues developed an electrochemical approach to metallic clusters deposited in situ on Au tips and electrodes, including Cu, Ag, Pb, and Pd (Figure 4E,F) [26,27,66]. The clusters contained approximately 3–4 layers and tens of metallic atoms. This approach extended the available electrode materials that would otherwise be subjected to oxide formation under ambient conditions. For junctions of α,ω-alkanoic acids (HO_2_C-(CH_2_)*_n_*-CO_2_H, *n* = 1–5) and under electrochemical control, G*_n_*_=0_ on Cu clusters was found to be larger than that on Ag clusters [27,66]. The authors considered that the difference between the β*_n_* values of 0.95 (± 0.02) for Cu and 0.71 (± 0.03) for Ag was small and that the junction conductance was mainly determined by the carboxylate–electrode coupling [27,66]. Later, on electrodes of Pd clusters, an even smaller β*_n_* value of 0.652 was obtained [59]. To further examine the peculiar β*_n_* values, instead of clusters, bulk Ag, Cu, and Pd electrodes were prepared, and BJ measurements were carried out in air [67]. The conductance in air for Cu electrodes was unavailable due to oxide formation. For Ag and Pd, the β*_n_* values measured in air were 1.0 and 1.1, respectively. These values agreed well with the values reports from the literature that were measured without an electrochemical control [27,66]. After considering the electrochemical potentials of the electrodes, the barrier heights formulated by Φ_B_ (Φ_B_ = E_F_ − E_HOMO_) correlated well with the values of β*_n_* obtained on the potentiostatted Pd, Ag, and Cu clusters, thus showing the importance of experimental environments and applied conditions.

Venkataraman and co-workers reported a method of fabricating a single-molecule junction based on Au−C contacts. The model molecules were those terminated with trimethylstannyl (−Sn(CH_3_)_3_) [68,69] and iodine headgroups (−I) [70]. In BJ experiments, these headgroups were found to be spontaneously detached from the molecules, resulting in Au−C contacts. The authors showed that −Sn(CH_3_)_3_-terminated alkanes gave a junction conductance that was two orders of magnitude higher than that of their amine-terminated analogues [68]. Schroeder and co-workers reported the formation of covalent contacts on Au and Ag electrodes by using acetylene-terminated oligophenylenes (HCC-(C_6_H_4_)*_n_*-CCH, *n* = 1–3) (Figure 4G) [71]. The junction conductance on Ag electrodes was about 10 times larger than that on Au electrodes. The conductance discrepancy was ascribed to the difference in binding configurations based on the analysis of computational modeling.

### 3.2. Fe, Co, and Ni Electrodes

Fe, Co, and Ni are magnetic materials with an unequal density of states (DOS) for the spin-up and spin-down electrons. When a molecule is bridged between electrodes of these metals, the effects of surface adsorption on the molecule are spin-dependent. Specifically, the degrees of energy-level shifts (Δ) and broadening (Γ) depend on the direction of the spin, which is associated with the DOS of the electrodes. Hence, the electron transport across such EME junctions is spin-polarized, and its behavior can be further engineered via the rational design of magnetic molecules. The advancement in spin-polarized transport across molecular thin films (also termed molecular spintronics) [72] expands our understanding of single-molecule junctions.

**Figure 5:**
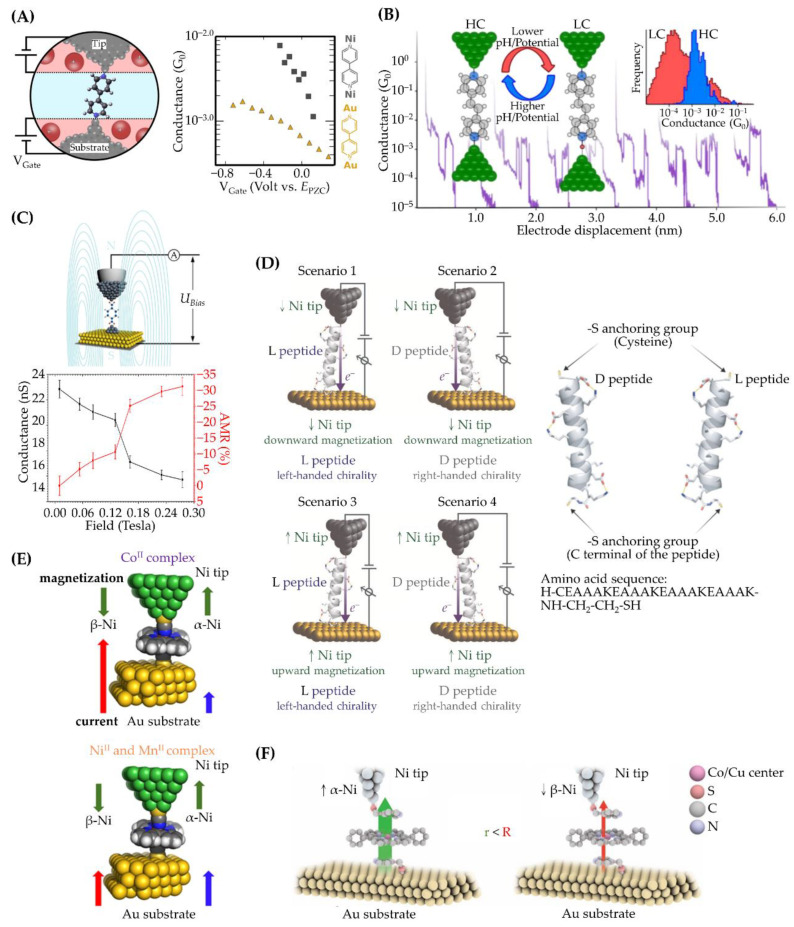
Schematic illustrations of representative research on investigating single-molecule conductance on magnetic electrodes. Electrodes: (**A**) Ni and Au; (**B**) Ni; (**C**) Fe clusters prepared with the approach of jump-to-contact EC-STM; (**D**–**F**) a pair of a Ni tip and Au substrate. (**A**) A 4,4-bipyridine junction under electrochemical control. The applied V_Gate_ is plotted against the potential of zero charge (PZC) of the Ni and Au substrates, which was −0.87 V or +0.13 V versus a saturate calomel electrode (SCE), respectively. The molecular conductance on Ni electrodes was found to be higher than that on Au electrodes. (**B**) Two sets of conductance histograms for 4,4′-vinylenedipyridine. In addition to the applied electrochemical potential, the junction conductance is sensitive to the solution acidity, which is ascribed to protonation/deprotonation of vinylenedipyridine. (**C**) Conductance measurement of terephthalic acid with electrodes of Fe clusters. The dependence of the junction conductance on the external magnetic field shows the behavior of anisotropic magnetoresistance. (**D**) Illustration of spin-polarized transport regulated by α-helical peptides with two isomeric forms: dextrorotatory (D-) and levorotatory (L-). With the same bias direction, the four scenarios of arrangements confer the descendant order in conductance of 1 > 4 > 3 > 2. (**E**) EME junctions of Ni-[M(tzpy)_2_(NCS)_2_]-Au (M = Mn, Ni, Co; tzpy = 3-(2-pyridyl)-[1,2,3]triazolo[1,5-α]pyridine). The transport is spin-dependent for Co(tzpy)_2_(NCS)_2_ junctions, yet spin-independent for Ni and Mn complexes, suggesting that the metal center plays an essential role. (**F**) Spin-dependent junctions involving supramolecular assemblies of a metalloporphyrin sandwiched between pyridine-4-yl-methanethiol molecules. The figures are adopted from [20,74,75,79,80,81].

Schwarzacher and co-workers employed an EC-STM to study the conductance of 4,4′-bipyridine between an EC-etched Ni tip and a ~100-nm-thick Ni film on Au substrates (Figure 5A) [20]. To provide oxide-free Ni electrodes, the tip was subjected to a negative potential to reduce the oxides, and the Ni substrate was prepared via in situ electrodeposition in the EC-STM cell to avoid nickel oxide formation. The snapback spacing for Ni electrodes was determined to be 2.5 Å. To afford the electrodes a constant magnetic field, an electromagnet with a 2 kOe magnetic field was placed underneath the substrate. The Ni–bipyridine–Ni junction exhibited only one conductance distribution [20], which was distinct from the two conductance peaks obtained for Au electrodes, where the high and low values corresponded to π- and a σ-dominant binding geometries [73] with the molecule tilted and vertical to the substrate, respectively. The authors carried out DFT-based calculations with and without considering spin polarization. The experimental findings did not agree with the non-spin-polarized calculations, which suggested that there were two conductance peaks with a vertical orientation that was more than three times that of the tilted one. Spin-polarized calculations predicted one conductance peak, which arose from the minority spin channel in which the FMO (specifically, LUMO) of bipyridine interacted strongly with the d bands of Ni electrodes at E_Fermi_. For the majority spin channel, the corresponding transmission peak resulted from the hybridization of the molecular HOMO and was relatively far from E_Fermi_ such that the contribution to the conductance was insignificant. This example demonstrated the importance of spin dependence for the discussion of junction currents flowing across Ni electrodes. This study also found that the EC gating on Ni electrodes was more efficient than that on Au electrodes in the examined potential range, where FMO pinning on Au was proposed to counteract EC gating. By taking advantage of the gating efficiency, this EC-STM scheme was further applied to configure Ni-4,4′-vinylenedipyridine-Ni junctions and to show conductance switching in simultaneous response to the solution pH and EC gating (Figure 5B) [74].

Mao and co-workers [75] prepared junctions of Fe-cluster electrodes in an ionic liquid that contained FeCl_3_ and the target molecule, terephthalic acid (TPA). To fabricate the junctions, Fe was electrochemically deposited on an STM tip, which was then brought toward the Au(111) substrate. A jump-to-contact event [26] would take place and transfer Fe atoms from the tip to the surface of Au(111). Subsequently, the retraction of the tip would leave an Fe cluster on the substrate. The direction of the external magnetic field was set to be perpendicular or parallel to that of the junction current (Figure 5C). For the perpendicular direction, the conductance of Fe–TPA–Fe junctions was not affected by field strengths of up to 0.300 T. For the parallel direction, a larger magnetic field or a smaller bias voltage resulted in a smaller junction conductance. With a parallel magnetic field strength of 0.230 T and a tip-biased voltage of −50 mV, the Fe–TPA–Fe junctions exhibited a giant anisotropic magnetoresistance of up to 53%, which was calculated with $(G_{⊥}-G_{∥})/G_{⊥}$, where $G_{⊥}$ and $G_{∥}$ are the molecular conductance measured at the molecular junction perpendicular and parallel to the magnetic field, respectively.

### 3.3. Junctions of Ni-Au Pairs

The spin-dependent studies introduced in Section 3.2 involved EME junctions with two ferromagnetic electrodes and a diamagnetic molecule. In this section, one electrode is a magnetized Ni tip and the other one is Au. The molecules act as a spin filter, and they contain a metal center with unpaired electrons. For an atom with a large atomic number, its electrons experience strong spin–orbit coupling, which generates a magnetic field with the orbiting electrons due to the motion of spin and orbiting. Considering the symmetry breaking of the crystal potential, the spin–orbit coupling leads to so-called Rashba effects, which were first reported in Au(111) by Jensen and co-workers [76]. The characterizations with photoemission spectroscopy demonstrated that the band structure of the *sp*-like surface states was spin-dependent [76,77,78]. This feature suggests that even though Au is not a conventional magnetic metal, it can act as a model surface for investigating the electron transport associated with the spin filter effect due to its spin polarization and the splitting of the surface density of states (DOS).

Mujica and co-workers studied the conductance of an α-helical peptide sequence bridged in a Ni tip and an Au substrate via STM-BJ (Figure 5D) [79]. The Ni tips were magnetically polarized ex situ before measuring the junction conductance. The results showed features of the spin-dependent transport for EME junctions of the L- and D-isomers. The conductance order of the L(D)-isomers was reversed as the magnetization direction of the Ni tip was switched. Such a reversed order in conductance magnitudes was also found upon changing the biasing direction. Specifically, for downward-magnetized Ni tips and electrons being transported from the tip (substrate) to the substrate (tip), the conductance of L(D)-isomers was larger than that of their counterparts, suggesting that the chirality of the peptide had a favored polarization direction of the injected downward (upward) spin from the Ni tip. Hence, the conductance of the parallel configuration (i.e., the polarization of the injected spin was the same as that favored by the peptide) was higher than that of the anti-parallel configuration (i.e., the polarization of the injected spin was opposite to that favored by the peptide). The conductance discrepancy between the two conditions of parallel and anti-parallel configurations was ascribed to the spin-polarized DOS on the Au substrate.

The groups of Ruiz and Diez-Perez employed the STM-BJ technique to measure the conductance of a series of complex molecules ([M(tzpy)_2_(NCX)_2_], where M = Mn, Ni, Co; tzpy = 3-(2-pyridyl)-[1,2,3]triazolo[1,5-α]pyridine; NCX = NCS or NCSe) bridged in Ni tips and Au substrates (Figure 5E) [80]. The Ni tips were magnetically polarized ex situ before the STM-BJ experiments. For Mn^II^ and Ni^II^ complexes, the molecular conductance was found to be independent of the polarized direction of the Ni tips, while a dependency was observed for the Co^II^ complexes. This was well explained by their ELA degrees, which were derived from spin-polarized calculations. The energy levels of FMOs for the Mn^II^ and Ni^II^ complexes were far away from E_Fermi_, yet close for the Co^II^ complexes. To investigate the effect of the substrate, the conductance of the Co^II^ complexes on Cu and Pt substrates was examined. A spin-dependent behavior and, thus, magnetoresistance were not found on these substrates. For the Cu substrate, it is diamagnetic, and thus, the spin–orbit coupling was not relevant. For Pt, although it had a large atomic number, its surface DOS was not dominated by s- and p-orbitals. Accordingly, the Rashba effect was insignificant on these substrates, suggesting that their surface DOS would not be spin polarized. With the support of theoretical calculations, the magnetoresistance on the Ni/Co^II^ complex/Au junctions was ascribed to the asymmetric coupling between the FMO and the spin-polarized surface DOS due to the strong spin–orbit coupling of Au. This work revealed that, in addition to the magnetism of the molecule and the ELA degree, the spin–orbit coupling effects of the substrate may play an essential role in magnetoresistance.

This group also investigated spin-polarized transport across a single supramolecular junction (Figure 5F) [81]. The supramolecules were a series of metalloporphyrins (M(DPP), where M = Co, Ni, Cu and Zn; DPP = 5,15-diphenylporphyrin) sandwiched between two pyridine-4-yl-methanethiol molecules. Three conductance sets were found for these supramolecular junctions bridged between the Ni tip and the Au substrate. The highest conductance set was ascribed to the metal center and spin polarization of the Ni tip. The values of the highest conductance for the Co and Cu complexes depended on the magnetization direction of the Ni tip. This feature was not observed for Ni and Zn complexes. This discrepancy was ascribed to the electronic configurations of the molecules. Specifically, NiDPP in the EME junction had a small energy difference between S = 1 (high spin) and S = 0 (low spin), while ZnDPP was a diamagnetic complex. The FMOs of the Co and Cu complexes were spin-polarized, and they were associated with the transport pathway.

### 3.4. Bimetallic Electrodes

**Figure 6:**
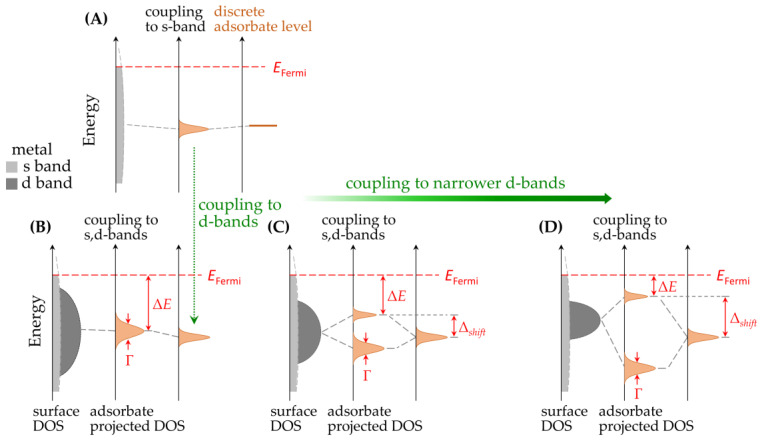
Proposed improvement of energy-level alignment (Δ*E*) with narrow surface d-bands on a bimetallic surface. (**A**) The adsorbate state is broadened due to its interactions with the surface s-states. (**B**–**D**) The broadened state is (**B**) further broadened or (**C**,**D**) split into a bonding state and an anti-bonding state due to its interactions with the surface d-states. Whether it develops into band broadening or splitting depends on the distribution of surface d-states (or d-bands). Additionally, the narrower surface d-bands may drive a shift of the anti-bonding state closer to the Fermi level, resulting in a better degree of ELA. This figure is adopted from [57].

The preparation of multi-component electrodes is an approach to tuning interfacial properties via molecule–electrode contacts. When a foreign metal adatom is introduced onto a metallic substrate, the discrepancy of the electron affinities drives a partial charge transfer, which reshapes the electron density and, hence, the surface band structure. This is a guiding principle for the interactions between a molecule and an electrode and is known as Norskov’s d-band model [31,32]. Based on this model, Chen and co-workers demonstrated the advantages of utilizing bimetallic materials as a new type of electrode (Figure 6) [57]. The bimetallic electrodes were Au substrates modified with a one-atom-thick Ag or Cu adlayer [57] via an electrochemical phenomenon called underpotential deposition (UPD), which limited the electrodeposited metals to one or two atomic layers [82]. The molecular conductance of α,ω-alkanoic acids (HO_2_C-(CH_2_)*_n_*-CO_2_H; *n* = 4, 6, 8) on these bimetallic electrodes was found to be 40–60 times higher than that on bare Au electrodes. Furthermore, this concept was extended to bimetallic electrodes with a Pt or Pd adlayer on Au and showed a higher junction conductance than that of bare Au [54]. According to the d-band model, the improved conductance was associated with the modified Δ, Γ [57], and surface density of states [54], suggesting that the surface modification of electrodes is a practical approach to tuning their interfacial properties.

## 4. Carbon-Based Material Electrodes

**Figure 7:**
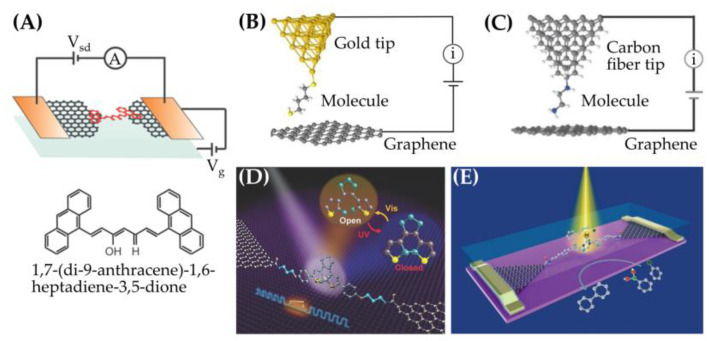
Recent advances in carbon-based electrodes for single-molecule junctions. (**A**) A single-molecule transistor in which the source and drain are a pair of graphene electrodes prepared via feedback-controlled electroburning. The model molecule was (1,7-(di-9-anthracene)-1,6-heptadiene-3,5-dione, and the molecule–electrode contact was made by π-π stacking through the anthracene moiety. (**B**,**C**) Single-molecule junctions of α,ω-alkanes on graphene electrodes. The junctions are formed with the STM i(s) mode. The tips in panels B and C are gold and carbon fiber, respectively. (**D**) A diarylethene molecule covalently bonded to graphene electrodes. The molecule transformed between the open and closed forms under visible and ultraviolet irradiation, respectively, exhibiting the electric behavior of photoswitching. (**E**) A single-molecule catalyst containing a Pd center is covalently bridged in a pair of graphene electrodes. The Pd complex activates the Suzuki–Miyaura cross-coupling reaction. By monitoring the current across the junction, the reaction mechanism and the associated kinetic and thermodynamic information are derived. The figures are adopted from [7,83,84,85,86].

Carbon-based electrodes, such as those containing graphene and carbon nanotubes, are important materials for the fabrication of single-molecule junctions because of the following advantages. Firstly, single molecules can bridge the termini of carbon-based electrodes via covalent bonds or non-covalent π-π stacking interactions. Secondly, carbon-based electrodes are easily prepared on a large scale. Last, carbon-based electrodes feature unique electronic properties associated with their shapes or sizes. Van der Zant and co-workers demonstrated an electroburning approach for fabricating a gap with a separation of 1–2 nm between two graphene electrodes on which anthracene-terminated curcuminoid molecules (1,7-(di-9-anthracene)-1,6-heptadiene-3,5-dione) were deposited (Figure 7A) [83]. The molecules were coupled with the electrode through π-π stacking between the anthracene-based headgroup and the electrodes. Gate-tunable *i*-V characteristics can be delivered by such molecular junctions.

Nichols and co-workers employed i(s) techniques to characterize the conductance of a series of α,ω-alkane dithiols (HS-(CH_2_)*_n_*-SH, *n* = 4, 6, 8, 10, 12) that were unsymmetrically bridged between an STM tip and graphene (Figure 7B) [84]. The tunneling decay constant (β*_n_* = 0.40 per methylene group) of Au–molecule–graphene was found to be smaller than that of Au–molecule–Au junctions (β*_n_* ~ 0.90). For the unsymmetrical EME junctions, the weak thiol–graphene contact and the strong thiol–gold coupling enhanced the dipole formation at the junction. The HOMO in the asymmetric junction was shifted toward the Fermi level, leading to a better degree of ELA.

Yang and co-workers investigated the conductance of a series of α,ω-alkanes (X-(CH_2_)*_n_*-X, X = −SH, −NH_2_; *n* = 2, 4, 6, 8, 10) bridged between a carbon fiber and graphene electrode (Figure 7C) [85]. The β*_n_* values were 0.23–0.39, which were comparable to those of asymmetric Au–molecule–graphene junctions, suggesting that graphene electrodes are a promising platform for molecular devices. Guo and co-workers developed a photosensitive device prototype in which a single diarylethene molecule was covalently bonded between graphene electrodes (Figure 7D) [86]. Under the irradiation with UV/visible light, the diarylethene molecule transformed between two isomeric forms carrying different electrical conductance. This device exhibited a high ratio of on/off currents (up to 100) and operated over a long-term period (10^5^–10^6^ cycles), demonstrating its excellent reproducibility as an optical switch. This group also fabricated a single-molecule-based Pd catalyst that was covalently bonded between graphene electrodes (Figure 7E) [7]. The catalyst contained a Pd center ligated with an *N*-heterocyclic carbene and activated the Suzuki–Miyaura cross-coupling reaction. The electrical currents across the single-molecule device were dependent on the intermediates of the Pd catalyst in each catalytic step. Accordingly, one could monitor the conductance of the device to identify the full cycle of the catalytic process.

## 5. Concluding Remarks

To summarize, more research efforts are engaged in moving the single-molecule electronic devices forward to the practical level. Molecular junctions with a high degree of robustness are required to achieve this objective. Hence, the fine-tuning of molecule–electrode interfacial properties and the understanding of their underlying science are essential. Herein, two electrode materials were discussed, namely, metallic and carbon-based materials. For the first one, with the BJ technique, EME junctions are ideal platforms for discovering emergent phenomena of electron transport at the nanoscale. One can explore their potential applications and verify their prospective concepts. The primary challenge that of making the fabrication of EME junctions reach the industrial level. For the second one, carbon-based EME junctions are underexplored systems in the field of molecular electronics. Carbon-based EME junctions feature interfacial contacts with covalent bonding, demonstrating that they are potential candidates for molecule-based electronic devices with robustness. Their functionalization is expected to be realized in combination with the highly developed techniques of organic synthesis. Additionally, it is prospective to investigate molecular junctions based on other emergent 2D materials, such as transition metal dichalcogenides, hexagonal boron nitride, and the derivatives of their heterostructures.

## Figures and Tables

**Table 1:** Examples of molecule–electrode interfacial properties for single-molecule junctions of α,ω-alkanes (X-(CH_2_)*_n_*-X).

Headgroups (X)	Electrode Materials	*n*	Junction Conductance (×10^−3^ G_0_)	G-Ratio (vs. G_Au_) ^a^	E_FMO_ (eV) ^a^	Γ (meV) ^a^	Rupture Force (nN) ^b^	Mechanism	Refs. No.
−SH	Pt	8	0.48	1.85	---	---	---	coupling	[50]
−SH	Au	8	0.26	1	−0.98	14.88	1.50	---	[49,50]
−SMe	Au	6	0.19	1	−0.91	12.9	0.42 (±0.22)	---	[54]
−SMe	Au	4	1.4	1	---	---	0.70	---	[51]
−NH_2_	Ag	4	0.26 ^c^	0.29	---	---		ELA	[55]
−NH_2_	Au	4	0.9 ^c^	1	---	---	0.69	---	[55,56]
−CO_2_H	Ag	6	0.018	2	−0.99	3.43	0.83 (±0.30)	ELA	[57]
−CO_2_H	Au	6	0.009	1	−0.96	2.73	0.60 (±0.30)	---	[57,58]
−CO_2_H	Pd	2	0.30	4.17	---	---	---	coupling	[59]
−CO_2_H	Fe	2	0.0065	0.54	---	---	---	---	[60]
−CO_2_H	Ag	2	0.17	2.36	---	---	---	coupling	[27]
−CO_2_H	Cu	2	0.23	3.25	---	---	---	coupling	[27]
−CO_2_H	Au	2	0.072	1	---	---	---	---	[27]
−CN	Pd	6	0.12	1.09	---	---	---	coupling	[36]
−CN	Pt	6	0.29	2.64	---	---	---	coupling	[36]
−CN	Au	6	0.11	1	0.69	6.1	0.43 (±0.40)	---	[36,54]
−NCS	Pd	6	0.59	2.95	---	---	---	coupling	[50]
−NCS	Pt	6	0.73	3.65	---	---	---	coupling	[50]
−NCS	Au	6	0.20	1	---	---	0.69	---	[50]

^a^ Notation: E_FMO_: energy level of the frontier molecular orbital determined via a single-level model or TVS; Γ: molecule–electrode couplings determined via a single-level model or TVS; G-ratio: the ratio of the measured conductance to that on bare Au electrodes; ---, not available. ^b^ Measured via AFM. ^c^ These values are not explicitly reported in the text of the literature but were estimated from the conductance histograms or summarized plots.

## Data Availability

No new data were created or analyzed in this study. Data sharing is not applicable to this article.
